# Coronavirus Disease Transmission Prevented in an Endoscopy Unit with Universal Protective Measures and No Systematic Preprocedural Testing

**DOI:** 10.3390/jcm11061681

**Published:** 2022-03-18

**Authors:** Lucía Guilabert, José Ramón Aparicio, Lucía Medina-Prado, Juan Carlos Rodríguez-Díaz, María Luisa Gomis, Pablo Chico-Sánchez, José Sánchez-Payá, Rodrigo Jover

**Affiliations:** 1 Gastroenterology Department, Instituto de Investigación Biomédica ISABIAL, Hospital General Universitario de Alicante, 03010 Alicante, Spain; lucia.guilabert@gmail.com (L.G.); japariciot@gmail.com (J.R.A.); lucia.medinaprado@gmail.com (L.M.-P.); gomis_mar@gva.es (M.L.G.); 2 Microbiology Derpartment, Instituto de Investigación Biomédica ISABIAL, Hospital General Universitario de Alicante, 03010 Alicante, Spain; rodriguez_juadia@gva.es; 3 Preventive Healthcare Department, Instituto de Investigación Biomédica ISABIAL, Hospital General Universitario de Alicante, 03010 Alicante, Spain; chico_pab@gva.es (P.C.-S.); sanchez_jos@gva.es (J.S.-P.); 4 Clinical Medicine Department, Faculty of Medicine, Universidad Miguel Hernández, 03202 Elche, Spain

**Keywords:** coronavirus, COVID-19, endoscopy, SARS-CoV-2

## Abstract

Background and aims: Even after two years of pandemic, there are still uncertainties on how to proceed when we schedule endoscopic procedures. During the COVID-19 pandemic, some scientific societies recommended universal preprocedural testing for all patients. However, other societies recommended against and considered enough to maintain strict infection control strategies. Our aim was to evaluate this approach in order to see if it was safe for both patients and healthcare workers to proceed with the endoscopies without performing a systematic PCR on all patients. Methods: Retrospective chart review of all patients undergoing endoscopy without preprocedural COVID testing at our center from March 2020 to May 2021. PCR tests performed in the patients receiving an endoscopic procedure were analyzed, and patients who tested positive between 14 days before and after the endoscopic procedure were selected. The registry of the endoscopy unit members participating in these procedures was also analyzed. Results: A total of 10,132 procedures were performed in the unit with 26 patients infected with SARS-CoV-2. Nineteen of these procedures were performed in patients with unknown SARS-CoV-2 carrier status. In 23 (88.5%) cases, transmission occurred through social or familial contact, and in 3 (11.5%), transmission occurred in the hospital. Four health care workers became infected during this period and none of them were related to the endoscopic procedures performed in patients with COVID-19. Conclusions: SARS-CoV-2 positive testing in asymptomatic ambulatory patients is rare and the adequate use of individual protective measures emerges as the main way to control the spread of COVID-19 infection in endoscopy centers.

## 1. Introduction

The World Health Organization (WHO) declared the SARS-CoV-2 coronavirus pandemic on 11 March 2020. Due to restrictions imposed during the coronavirus disease 2019 (COVID-19) pandemic, activity in endoscopy units was nearly completely stopped in April-May 2020 in Spain to reduce the risk of contracting the infection and help decrease its spread. Procedures were prioritized based on the scientific society’s guidelines, and in some cases, only emergent/urgent procedures were performed [1,2,3]. In reopening endoscopy units, some scientific societies recommended universal, preprocedural testing for all patients scheduled for endoscopic procedures because a large proportion of coronavirus infections is transmitted by asymptomatic individuals, particularly in high-prevalence areas [4,5,6,7]. However, other societies recommended against universal preprocedural testing; they considered the maintenance of strict infection control strategies sufficient and recommended the use of individual protective equipment for all personnel involved in the procedure [2,8]. The pandemic is still continuing around the world; in fact, there was a recent increase of the incidence in the last months, even in countries with high vaccination rates. Even after two years of pandemic and already six COVID waves, there are still uncertainties on how to proceed when we schedule endoscopic procedures and if it is necessary to perform systematic PCR or not. Due to that, we ran this retrospective study to evaluate the outcomes of this latter strategy (the use of protective equipment) in a tertiary endoscopy unit in an area with high COVID-19 prevalence in Spain, and see if it was safe, for both patients and healthcare workers, to proceed with the endoscopies without performing a systematic PCR on all patients, as it was established in our unit.

## 2. Methods

**Figure 1:**
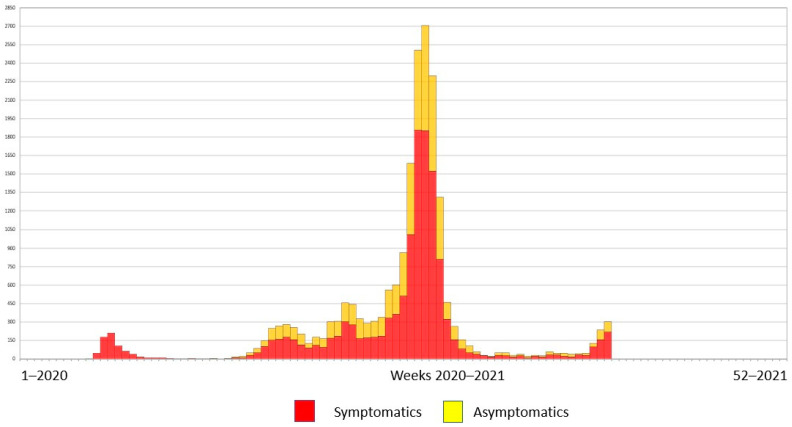
COVID-19 incidence in the Health Department of Alicante–Hospital General in 2020 and 2021. The 14-day COVID-19 incidence rose above 250 cases/100,000 inhabitants within 23 weeks, and above 500 cases/100,000 inhabitants within 8 weeks.

We conducted an observational retrospective study with the review of the medical charts of all patients that underwent endoscopies at the Hospital General Universitario de Alicante from 15 March 2020 to 9 May 2021. During this period, the Spanish government declared a state of national emergency. The 14-day COVID-19 incidence rose above 250 cases/100,000 inhabitants within 23 weeks, and above 500 cases/100,000 inhabitants within 8 weeks in the Health Department of Alicante–General Hospital where our endoscopy unit is located (Figure 1).

Preprocedural testing was not performed routinely for any of the analyzed endoscopic procedures. However, all patients underwent screening for COVID-19 symptoms prior to their endoscopy. Only patients with a clinical suspicion of COVID-19 underwent SARS-CoV-2 PCR testing, and the endoscopic procedure was postponed. We analyzed all SARS-CoV-2 PCR tests performed during the study period, for any reason, in patients that received an endoscopic procedure. We included in our study all patients that tested positive for SARS-CoV-2 PCR between 14 days before and 14 days after the endoscopic procedure. We selected this period of time because this is the possible infective phase of the virus. Positive cases were traced by a personal interview with affected patients and transmission origin being defined as socio-sanitary (unprotected contacts in the work environment, in the interrelationship between health professionals), social (out-of-hospital, in the usual living environment), and healthcare-associated infection (during patient care, in routine healthcare).

During the study period, 35 health professionals participated in the endoscopic procedures (18 endoscopists, 8 endoscopy nurses, and 9 nurse assistants). During the endoscopic procedures, all healthcare workers involved were required to wear individual protective equipment, including an FFP-2 mask, protective eyewear, gloves, a waterproof gown, and a surgical cap. We also accessed the registry of endoscopy unit members that participated in these procedures to identify those with COVID-19 infections. We analyzed the outcomes of these patients and investigated potential transmission to the health professionals involved in the procedure.

## 3. Results

During the study period, 10,132 procedures were performed in the unit: 7029 in outpatient settings and 3103 in inpatient settings. Based on the PCR tests, 26 patients were infected with SARS-CoV-2 PCR between 14 days before and 14 days after the procedure, and 27 endoscopy procedures were performed in these patients (one patient underwent two procedures). At the time of the endoscopy, during the potential transmission window, 8 procedures were performed in patients with already known positive COVID-19 test results, and 19 were performed in patients with unknown carrier status.

The characteristics of COVID-19 positive patients and the procedures performed are shown in Table 1. Most of them were outpatients (*n* = 17/26, 63%) and elective procedures (21 elective vs. 6 urgent/emergent). The indications for endoscopy were diverse, being the more frequent lower gastrointestinal bleeding (5/27, 18.5%) followed by colorectal cancer screening (4/27, 14.8%) and iron-deficiency anemia (3/27, 11.1%). No patient with COVID-19 developed any complications related to the endoscopic procedure. Although we do not have the individual data, during the study period, the variants that prevailed in our hospital were the alpha variant (B.1.1.7) and the European variant (B.1.177). The delta variant was first detected in our hospital in June 2021 and omicron in December 2021.

The preventive medicine department of our center evaluated potential mechanisms of transmission with a structured interview and contact study. In 23 (88.5%) cases, transmission occurred through social contact, and in 3 (11.5%) cases, the transmission was considered as healthcare-associated. All the healthcare-associated cases were transmitted from hospital roommates and were not related to the healthcare professional assistance.

During the study period, four healthcare workers in the endoscopy unit became infected with COVID-19. In all four cases, transmission occurred through social contact, unrelated to the endoscopic procedures performed in patients with COVID-19.

None of the patients infected with COVID-19 were vaccinated against SARS-CoV-2 at the moment of diagnosis. Also, none of the infected healthcare workers were vaccinated at that moment. In Spain, vaccinations were initiated on 8 January 2021 for healthcare workers, and in the month of February for the general population, prioritized by age.

**Figure 2:**
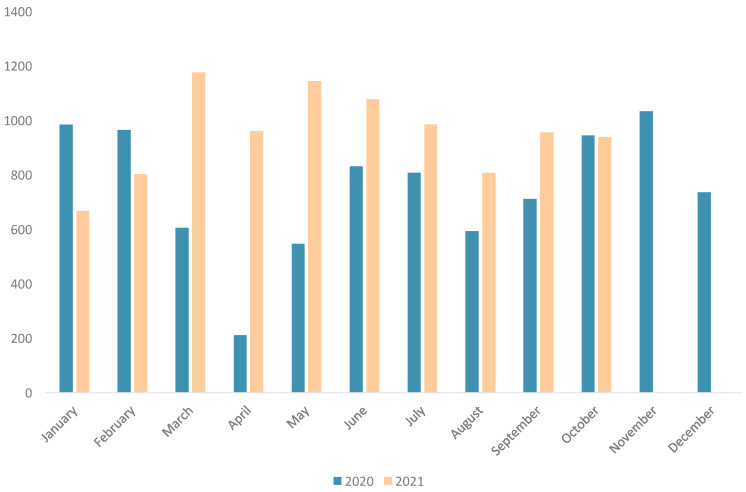
Number of endoscopic procedures performed in Alicante’s endoscopy unit in 2020 and 2021. Infection control strategy enable us to resume our pre-pandemic endoscopic activity rapidly.

The activity in our endoscopy unit was almost stopped at the beginning of the pandemic during the months of March and April; however, the number of examinations performed was rapidly increased after reopening as can be seen in Figure 2, reaching levels similar to the pre-pandemic time.

## 4. Discussion

Our results showed that SARS-CoV-2 was not transmitted in a tertiary endoscopy unit located in a high-incidence area when all individuals involved followed infection control guidelines. Out of 10,132 procedures, only 26 patients had positive SARS-CoV-2 test results in our unit during the potentially infective phase of the disease, and only 19 were unknown at the moment the procedure was performed. The data seem consistent with previous reports which detected an incidence of less than 1% of COVID-19 patients undergoing endoscopic procedures during the pandemic [9]. Moreover, we detected no transmission from these patients to the attending healthcare workers (four professionals were infected during the study period, but not related to COVID-19 cases). These results highlighted the importance of maintaining an infection control strategy as the primary method for avoiding nosocomial transmission to health professionals. Conversely, our results argued against the importance of routine preprocedural SARS-CoV-2 PCR testing for infection control. In fact, in the same period in our hospital, patients who underwent surgery were screened for COVID-19 with universal preprocedural PCR and the incidence of a positive test was 0.0022 among asymptomatic patients. No healthcare workers-associated infections were detected [10].

It is true that PCR is a test with high sensitivity and specificity to detect SARS-CoV-2 infection, and therefore, if performed systematically to all the patients scheduled for an endoscopic procedure, we can detect some asymptomatic patients, avoiding the exposure to healthcare professionals and the possibility of getting infected during the procedure. On the other hand, PCR is normally performed 24–48 h before the procedure, and it might give a false sense of safety. Moreover, patients could be positive in the moment of the endoscopy. Therefore, even with a negative SARS-CoV-2 PCR, the use of protective equipment is still necessary. Moreover, performing PCR on all patients implies more appointments for the patients (with absence in their daily life duties), medical costs, and bureaucracy to all the professionals related to this medical act. Systematic preprocedural PCR can become an unnecessary barrier for the restart of normal activity in endoscopy units.

Routine testing for SARS-CoV-2 infection prior to a medical intervention could have two objectives: (1) to protect patients with asymptomatic COVID-19 from potential complications related to the procedures that might worsen as the disease evolves; and (2) to protect health professionals from virus transmission from asymptomatic SARS-CoV-2 carriers. However, most endoscopic procedures are diagnostic or minimally invasive, and they do not increase the risk of pulmonary or thrombotic complications in patients with COVID-19. In fact, in our cohort, none of the COVID-19 patients who underwent an endoscopy had a worsening of their disease as related to the procedure. Moreover, our results showed that health professionals were sufficiently protected by enforcing the rigorous use of individual protective equipment during endoscopy procedures. Indeed, endoscopy is an aerosolizing procedure [1], and SARS-CoV-2 RNA has been detected in the feces of patients with COVID-19 [11]. Thus, both upper and lower gastrointestinal endoscopic procedures are considered high risk for COVID-19 transmission. Consequently, the use of individual, effective protective measures is mandatory. It is easy to comply with these measures because they are very similar to the measures routinely used in endoscopy for many years.

Limitations of our observation are the retrospective nature of data collection and also that the data are coming from only one center. However, it is difficult to organize a randomized study including procedures with and without the use of preprocedural testing. Moreover, we believe that our data are extensive to other similar centers because, as has been said, protective equipment is very close to the measures usually employed in endoscopy units. We have not accounted for potential asymptomatic patients or healthcare workers that could participate in the described procedures. However, that did not have any repercussion in the analyzed data. We also excluded patients that when arrived at our endoscopy unit were symptomatic (fever, cough, or other symptoms). It was a simple screening performed by our nurses before the procedure, and as we did not perform the endoscopy of these patients, we might have lost some information in this setting. However, what we are trying to analyze is the necessity of a screening PCR for asymptomatic patients, and it does not affect the results of our study.

On the other hand, the main strength of our study is the large number of patients included, which allows us to obtain robust assurance of the safety of the described strategy.

In a large series of more than 10,000 procedures, we showed that the strict observance of protective measures was sufficient to prevent COVID-19 transmission. This result argued against the need for universal routine pre-endoscopy testing proposed by some scientific societies [4,5,6,7]. In a recent update, the American Gastroenterological Association retracted this recommendation. However, their update continued to recommend preprocedural testing in some settings, but at the discretion of individual endoscopy centers [12]. Other scientific societies have not changed until now their recommendation of universal preprocedural SARS-CoV-2 PCR testing and, in a recent update, ESGE still recommends that all patients presenting for GI endoscopy be required to provide either: a negative viral test performed within 48 h before their scheduled GI endoscopy or documentation of full COVID-19 vaccination status or recovery from COVID-19 infection within the past 6 months [13]. Our results show that this routine preprocedural testing is not justified, not providing additional protection for patients or healthcare workers, and these figures should be taken into account for potential new pandemic waves. Moreover, routine preprocedural SARS-CoV-2 PCR testing is expensive and cumbersome in daily practice, hindering the access of patients to our endoscopy units and also overloading already exhausted microbiology services. Finally, asymptomatic ambulatory patients rarely test positive for SARS-CoV-2 [9,14]. Thus, we argue that the routine use of individual protective measures is an adequate primary method for controlling the spread of COVID-19 in endoscopy centers, even in largely unvaccinated and high incidence settings.

The COVID-19 pandemic has challenged the provision of routine health care, resulting in a temporary curtailment of endoscopic activities [15]. In this context, universal preprocedural testing is another barrier for recovering after a backlog of endoscopy services. This strategy of not using systematic preprocedural SARS-CoV-2 PCR testing has allowed us to rapidly recover the endoscopic activity to pre-pandemic levels. Nowadays, when we can still find many countries or regions where the percentage of vaccinated people is low and the incidence is growing again, these results show that adequate implementation of universal protective measures is enough to reduce the spread of the virus, allowing handling the ordinary endoscopy burden without the need to reduce the activity in our endoscopy units.

## Figures and Tables

**Table 1:** Characteristics of patients with COVID-19 and the applied endoscopic procedures.

Case Number	Patient Age	Indication	Procedure	Date of Endoscopy	Time from Endoscopy to Positive SARS-CoV-2 PCR Result (Days)	Inpatient or Outpatient	Emergency or Elective Procedure
1	58	Lower GI bleeding	Colonoscopy	12 March 2020	9	Outpatient	Elective
2	96	Lower GI bleeding	Colonoscopy	24 October 2020	5	Inpatient	Elective
3	57	Epigastric pain	Gastroscopy	5 November 2020	2	Outpatient	Elective
4	50	CRC screening	Colonoscopy	9 December 2020	4	Outpatient	Elective
5	76	Jaundice	ERCP + EUS	11 December 2020	10	Inpatient	Elective
6	72	Abdominal mass	Colonoscopy	18 December 2020	2	Outpatient	Elective
7	66	CRC screening	Colonoscopy	21 December 2020	6	Outpatient	Elective
8	71	Lower GI bleeding	Colonoscopy	23 December 2020	11	Inpatient	Elective
9	61	Gastric volvulus	Gastroscopy	7 January 2021	4	Inpatient	Emergency
10	76	Gastric dysplasia	Gastroscopy	13 January 2021	7	Outpatient	Elective
11	53	CRC screening	Colonoscopy	14 January 2021	11	Outpatient	Elective
12	68	Gastric ulcer control	Gastroscopy	14 January 2021	12	Inpatient	Elective
13	57	CRC screening	Colonoscopy	25 January 2021	−14	Outpatient	Elective
14	54	Upper GI bleeding	Gastroscopy	30 January 2021	0	Inpatient	Emergency
15	62	Upper GI bleeding	Gastroscopy	28 January 2021	−3	Inpatient	Emergency
16	43	Vomiting	Gastroscopy	2 February 2021	1	Inpatient	Elective
17	77	Lower GI bleeding	Colonoscopy	4 February 2021	7	Outpatient	Elective
18	81	Anemia	Gastroscopy + colonoscopy	8 February 2021	−11	Inpatient	Elective
19	50	Acute cholangitis	ERCP	11 February 2021	−14	Inpatient	Emergency
20	74	Jaundice	ERCP	12 February 2021	−8	Inpatient	Elective
21	61	Variceal banding	Gastroscopy	17 February 2021	1	Inpatient	Elective
22 *	40	Acute cholangitis	ERCP + EUS	17 February 2021	0	Inpatient	Emergency
23	75	Anemia	Gastroscopy	25 February 2021	−4	Inpatient	Elective
24 *	40	Biliary prothesis removal	Gastroscopy	1 March 2021	−11	Outpatient	Elective
25	64	Lower GI bleeding	Colonoscopy	4 March 2021	2	Inpatient	Emergency
26	55	Vomiting	Gastroscopy	9 March 2021	−14	Inpatient	Elective
27	80	Anemia	Gastroscopy + colonoscopy	26 March 2021	−14	Inpatient	Elective

* Case numbers 22 and 24 were the same patient; this patient underwent two different procedures; GI: gastrointestinal; CRC: colorectal cancer; ERCP: Endoscopic Retrograde Cholangio-Pancreatography; EUS: Endoscopic Ultrasound.
